# Glowing Spicules and Structural Collapse: A Single-Cell Insight into the Oxidative Aging of Favism Erythrocytes

**DOI:** 10.3390/ijms27031132

**Published:** 2026-01-23

**Authors:** Giovanni Longo, Simone Dinarelli, Marco Girasole

**Affiliations:** Institute of Structure of Matter (ISM), Italian National Research Council (CNR), Via Fosso del Cavaliere 100, 00133 Rome, Italy; giovanni.longo@ism.cnr.it (G.L.); simone.dinarelli@ism.cnr.it (S.D.)

**Keywords:** erythrocytes, favism, cell aging, AFM, membrane roughness, nanomechanical properties

## Abstract

Erythrocyte aging is a fundamental physiological phenomenon that involves significant structural and nanomechanical alterations of the cells’ structure and function. Coupling optical, fluorescence, and Atomic Force Microscopy (AFM), we analyzed morphology, membrane roughness and nanomechanical properties of the very same RBCs arising from favism subjects, measured at different stages of their aging in vitro. We also investigated the evolution and abundance of vesicles arising from the cells over their senescence pathway. This approach combines high-resolution fluorescence imaging with the correlation of membrane topology and biomechanics. This explores the differences between investigation based on statistical morphometric parameters, such as membrane roughness, and those based on the measure of point-dependent nanomechanical properties. Our ultra-morphological study evidences the existence of clear differences in the aging of normal and favism erythrocytes that results in a larger number of cells with abnormal shapes and in a hyper-production of vesicles along the senescence pathway of favism cells. In explaining these differences, we focused on the roles played by the hemoglobin evolution and by the morpho-mechanical properties that are responsible for the skeletal alterations. In particular, our data reported evidence that the two corresponding degradative pathways are coupled and play an important enhancement role in promoting the progression of cell senescence.

## 1. Introduction

Erythrocytes, or red blood cells (RBCs), are fundamental parts of vertebrate life, since they are responsible for oxygen and carbon dioxide transport throughout the body. Their unique shape and remarkable membrane viscoelasticity allow them to flow in the blood vessels, traversing capillaries narrower than their own diameter [1,2]. This deformability is paramount for their function, a highly regulated property rising from the interaction between the lipid bilayer, the underlying spectrin-based cytoskeleton, and membrane-associated proteins. Any biochemical alteration that affects the structural integrity of the membrane skeleton can change RBCs’ mechanical and morphological properties, leading to hemodynamic consequences or even hemolysis in certain pathological conditions [3,4].

A prominent example of such a condition is favism, the most diffuse human enzymopathy, with a worldwide presence that has grown significantly due to population shifts and improved diagnostics [5,6]. This is a severe hemolytic anemia, which arises from a genetic deficiency in the Glucose-6-Phosphate Dehydrogenase (G6PD) enzyme and is triggered by exposure to specific oxidative stressors (most notably fava beans). According to recent epidemiological reviews, as of 2021, an estimated 443.3 million people (approximately 4.9% to 5.6% of the world’s population) live with G6PD deficiency globally, and approximately 16,700 to 33,000 deaths per year are attributed to this disease. While many individuals with the deficiency are asymptomatic, the global health loss, measured in Disability-Adjusted Life Years (DALYs—years of healthy life lost due to disability and premature death), is substantial. Approximately 679,000 DALYs are attributed to G6PD deficiency annually, with a significant portion coming from neonatal hyperbilirubinemia (jaundice) and its severe complication, kernicterus (brain damage from high bilirubin) [7].

The G6PD enzyme is critical for the pentose phosphate pathway, the sole source of nicotinamide adenine dinucleotide phosphate (NADPH), which is essential for maintaining the reduced state of glutathione (GSH), the cell’s primary antioxidant defense. In G6PD-deficient individuals, exposure to oxidative stress exhausts the limited antioxidant capacity, leading to oxidative damage to hemoglobin (Hb) and membrane components. This causes several pathological outcomes, including membrane stiffening, splenic sequestration, and intravascular hemolysis, as favism RBCs have a lesser resistance to osmotic or mechanical stresses [8,9,10].

While the biochemical cascade of G6PD deficiency is well-established, the nanostructural and nanomechanical consequences of this genetic defect on the erythrocyte membrane are less understood [11]. Literature data usually focuses on the behavior of the entire RBC populations or on the average metabolic response of the cells at the time of the investigation (conventionally, T0) and usually leads to the conclusion that favism RBCs have worse morphological and functional characteristics than normal cells [12,13]. However, recently, an approach based on the time evolution of normal and favism erythrocytes has been performed [14]. The study was based on a compared analysis of the ultra-morphological and biochemical properties of the normal and pathological cells during their accelerated in vitro aging.

The aging of RBCs is a basically important phenomenon with both physiological and pathological consequences. Indeed, as erythrocytes lack nucleic acids and cannot self-replicate, their turnover in the blood is ensured by a mechanism that relies on the recognition of cell senescence [15,16]. The aging pattern can be characterized as a sequence of intermediate states, distinct for morphological, structural, and functional properties [17,18]. Monitoring cell aging, thus, allows us to understand how a given sample is composed in terms of subpopulation and how those subpopulations evolve over time according to the environmental conditions and to the most critical biochemical parameters, including intracellular ATP [19].

This aging-based approach, applied to the comparison of normal and G6PD-defective RBCs, showed that understanding the properties of favism erythrocytes is more challenging than was expected. Indeed, G6PD-deficient cells evidenced specific metabolic and environmental adaptation mechanisms, which, remarkably, provide favism RBCs with a surprising degree of resilience to harsh conditions [14,20]. These findings suggest that the consequence of a G6PD deficiency does not induce just a simple, linear degradation of cell properties, but rather a complex adaptive process which takes place during the cell lifespan [21]. However, these new findings have remained largely at the population and at the biochemical-metabolic level, a circumstance that leaves open the question of what kind of structural alterations occur at the nanoscale and how the favism membrane evolves under the stress of aging.

The present work has the aim of analyzing the alterations in the cell properties induced by G6PD deficiency at the single RBC level. Many techniques have been used to monitor the diffusivity and deformation parameters relevant for the mechano-transduction processes in RBCs, including micropipette aspiration, magnetic twisting cytometry, or optical tweezers, which have yielded interesting results [22,23,24,25,26]. Among these tools, Atomic Force Microscopy (AFM), a powerful tool in nanomedicine and cell biology, seems to be particularly suited to perform nanoscale characterization of single RBCs: it provides quantitative topographical data with sub-nanometer resolution, from which statistical morphometric parameters such as the surface roughness, can be measured. Moreover, AFM can also probe the mechanical properties of a surface with similar high resolution, from which cell properties of the utmost relevance in physiological and pathological condition, such as the Young’s modulus, cell stiffness, adhesion, and deformation of the surface at the nanoscale, can be obtained [27,28,29,30].

We exploited these AFM peculiarities to compare the variations in the morphometric and nanomechanical properties observed during the artificial aging of erythrocytes from a G6PD-deficient patient. These results were complemented with fluorescence microscopy imaging, where the autofluorescence properties of hemoglobin were exploited to follow the evolution of these molecules at different aging times and in different conditions.

This study also aims to verify and improve our understanding of the adaptive mechanisms observed for RBCs from G6PD-deficient individuals by translating the biochemical-metabolic response to a structural and nanomechanical plane and to find a role for different cell components in the pattern of cell aging.

Our methodological framework couples nanomechanical mapping with a quantitative analysis of the membrane morphology, using the surface roughness as an indicator of the membrane’s structural arrangement, and we complemented this nanoscale approach with an overview of the alteration occurring in the auto-fluorescent properties of these cells. The membrane roughness, in particular, is an interesting morphometric parameter to study biosystems. In the case of RBCs, it couples the description of the membrane arrangement to a special sensitivity for the structural integrity of the underlying skeletal network, making it an ideal tool for a comparison with the pure nanomechanical detection of the cell’s properties [31,32]. Concerning the quantitative characterization of Young’s modulus, however, it is worth reminding that it is very much dependent on the experimental conditions (tip, environment, interaction forces, instrument parameters) and, chiefly, on the model chosen for the analysis of the force curves. As a consequence, for instance, the literature data reports values spanning several orders of magnitude for samples similar to that of our study [33,34,35,36,37,38]. This limitation in comparative analysis of samples, however, can be tackled by maintaining strict consistency in the measurement setup and analysis protocols among specimens.

In terms of methodology, our approach is innovative, since we performed a point-by-point correlation, along the cell aging, between the nanoscale morphometric arrangement of the membrane and the nanomechanical properties measured on the very same areas of the very same cells. This correlative methodology, recently tested in the case of erythrocytes, is very promising, as it allows not only the evaluation of average parameters on a population of cells, but also the exploration of the local heterogeneity of the membrane properties and the inter-dependency of its structure and function at the cell level.

To ensure robustness and comparability, we performed experiments on air-dried cells, since this sample preparation methodology is more widely diffused in the AFM community, permitting a more direct comparison with other works on RBC biophysics [39]. In particular, we monitored the in vitro aging, accelerated by maintaining the cells in starvation condition, and analyzed the erythrocytes characteristics in glass smears performed at different time points along the aging path. We focused on cell evolution in critical moments of the phenomenon, and for this reason we selected aging times of 1, 7, and 12 days that correspond to young, intermediate, and senescent RBCs.

By elucidating the nanoscale physical changes that accompany the aging of favism erythrocytes, this work aims to provide new insights into the pathophysiology of favism and the fundamental mechanisms governing RBC membrane stability under oxidative stress.

## 2. Results

**Figure 1:**
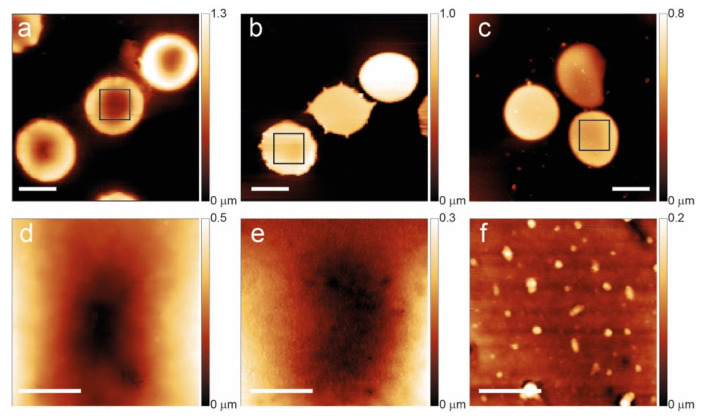
Aging path of normal RBCs at T1 (**a**,**d**), T7 (**b**,**e**), and T12 (**c**,**f**). The bottom panels evidence the membrane arrangement and the occurrence of vesicles in the squares of the upper panels. The scale bars for panels (**a**–**c**) are 5 μm while the scale bars for (**d**–**f**) are 1 μm.

**Figure 2:**
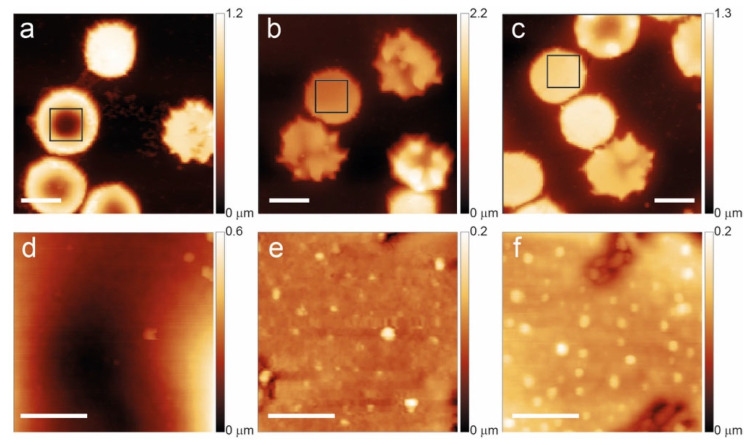
Aging path of favism RBCs shown at T1 (**a**,**d**), T7 (**b**,**e**), and T12 (**c**,**f**). The lower panels highlight the membrane arrangement and the occurrence of vesicles in the squares of the upper panels. The scale bars for panels (**a**–**c**) are 5 μm while the scale bars for (**d**–**f**) are 1 μm.

The glass smears bearing the RBCs were characterized using AFM at three specific time points: day 1 (T1), day 7 (T7), and day 12 (T12). Figure 1 and Figure 2 show the morphology of some of the investigated cells, showing the typical aging patterns observed, respectively, in control samples and in the cells of a subject affected by favism.

In the control sample, young cells show a typical biconcave shape with a homogeneous membrane structure, but the increase in aging leads to a morphological progression with the gradual development of abnormal shapes (flat and spiculated) and, at long aging times (T12), a significant formation of vesicles on the cell membrane.

In the case of favism, as shown in Figure 2, the aging process exhibits different characteristics. For instance, some morphological anomalies occur on a non-negligible fraction of cells even in young erythrocytes (T1). For instance, 16% of the observed cells have a spherocytic shape, and nearly 25% are spiculed. Moreover, the presence of vesicles on the membrane in the case of favism is much more pronounced and appears at shorter aging times (already common at T7 and systematic at T12) compared to the control.

Over time, the number of morphologically defective cells increases also in favism samples, but it is interesting to note that at intermediate and, especially, long aging times, the differences in overall cell morphology between control and pathological samples are smaller than those observed in young cells. For instance, the observed percentage of non-dyscocytic morphologies (spherocytes + spiculed cells) grows from less than 15% at T1 to nearly 50% at T12 in control samples, but they only increase from 42% to 53% in the corresponding favism samples. Yet, the different rate in the production and release of vesicle—which, it should be recalled, is inherently part of the aging pathway of RBCs and that in past years has been associated with erythrocyte-specific pathways of degradation (e.g., eryptosis) [40]—indicates that differences between the two samples are still in place at any time.

To better evaluate, on the nanoscale, the role of cell architecture in shaping the aging of normal and pathological cells, we measured the cell nanomechanical properties, and this data was compared with the information arising from a quantitative measurement of the plasma membrane roughness. Indeed, the methodology we employed in this analysis (see Section 4) allows comparing nanomechanical and morphometric data on the very same areas of the very same cells, enabling the comparison of these biophysical quantities both at the single-cell level and, by analyzing enough cells, also in terms of average values for a sample. These comparisons were performed at relevant points of the aging path.

**Figure 3:**
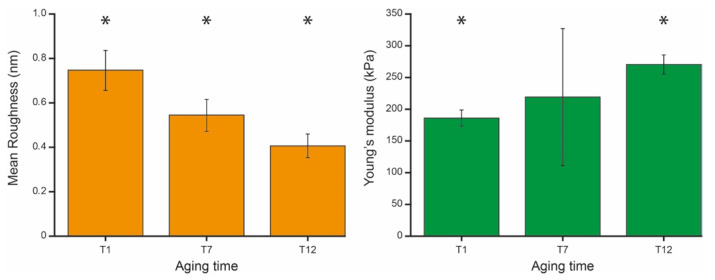
Average roughness (**left panel**, orange) and average Young’s modulus (**right panel**, green) measured at the chosen aging time. The values are reported as mean and standard deviation of the single cell’s mean values. The significance marks (*) indicate a statistically significant difference, with *p* < 0.001 calculated using a one-way ANOVA test.

Figure 3 shows the evolution over time of the mean roughness and mean Young’s modulus of the RBCs. These values were obtained by averaging on a given sample (e.g., aging time) the mean roughness values of the single cells that were calculated from the 500 × 500 nm^2^ square portions of the images.

Consistent with previously published works, the average roughness value decreases along the aging pathway [14].

The corresponding nanomechanical properties of the cells show an interesting pattern. As shown in Figure 3, the Young’s modulus increases along the aging in such a way that the cells result stiffer at the late stage of aging with respect to the initial phase. However, while there is a statistically significant difference between the mean values and standard deviations measured at T1 and at T12, at the intermediate aging time (T7) the Young’s modulus values are more spread and cannot be distinguished from younger and older cells. This result reflects the fact that, at an intermediate stage of aging, the RBCs’ mean mechanical properties are scattered. This means that, while in terms of roughness the entire population at T7 is quite similar, in terms of elasticity there are significant number of cells that still behave as young cells and others that already behave like old cells.

This interesting aspect can be further investigated. Indeed, the spatial resolution of the AFM is unique in its capability to collect both roughness and Young’s modulus maps of the cells, allowing the analysis of the variability of their values at a single-cell level.

**Figure 4:**
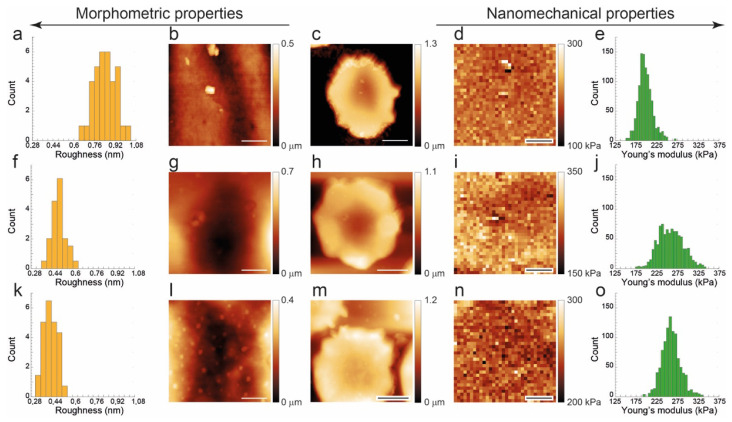
Roughness and elasticity mapping on cells with closely related, crenated, morphology collected at T1 (**top panels**), T7 (**middle panels**), and T12 (**bottom panels**). From left to right, the figure shows histograms of the roughness (**a**,**f**,**k**) calculated on the high-resolution topography reported in the side panels (**b**,**g**,**l**), low-resolution cell topography (**c**,**h**,**m**), Young’s modulus map acquired in the same region (**d**,**i**,**n**), histograms of Young’s moduli (**e**,**j**,**o**) collected on the single cells. Each high-resolution zoom-in for roughness determination was on a 4 × 4 microns image with 1000 points per line; each force-volume map contains 32 × 32 force curves collected on the very same 4 × 4 μm^2^ area. Scale bars are 1 μm in (**b**,**g**,**l**) and in (**d**,**i**,**n**) panels. Scale bars are 3 μm in (**c**,**h**,**m**) panels.

These results are shown in Figure 4, where we compare, on the very same cells, the roughness values (left) to the Young’s modulus values (right). The cells were chosen, at the different aging times, with very similar morphology to avoid large scale morphological effects in the comparison. For this reason, we selected three crenated cells. The rationale for this choice is that crenated cells are closely related to discocytic RBCs (i.e., they still have a good structural integrity) and, moreover, are the only cell types that can be easily found at all aging times.

The corresponding data is reported in Figure 4 and, although referring to the effects at the single-cell level, agree with the trend shown in Figure 3 for the entire samples: namely, the roughness decrease at increasing aging while the Young’s modulus increases. However, a significantly larges spread of the Young’s modulus histogram was observed in the cell at T7. Remarkably, such an effect cannot be reproduced in the histogram of the roughness calculated on the very area of the very same cell. In other words, the inhomogeneity detected in the nanomechanical properties is not reflected in membrane arrangements.

Since favism erythrocytes are overexposed to oxidative stresses due to their limitation in the reducing power production chain, a circumstance that is expected to have consequences at different levels in the cell structure and function, we focused our investigation on the role of Hb, which is the basic functional protein in these cells but can also play an important structural role. In the general landscape of the aging-related cell degradation, the hyper-oxidation of Hb in favism cells can significantly enhance the importance of the pathways associated with this fundamental protein.

To better understand these aspects, we analyzed the samples by fluorescence microscopy. The presence of Hb bestows RBCs with self-fluorescence properties, allowing a straightforward mapping of the time evolution of this protein, and making fluorescence microscopy a particularly powerful tool to characterize these cells.

**Figure 5:**
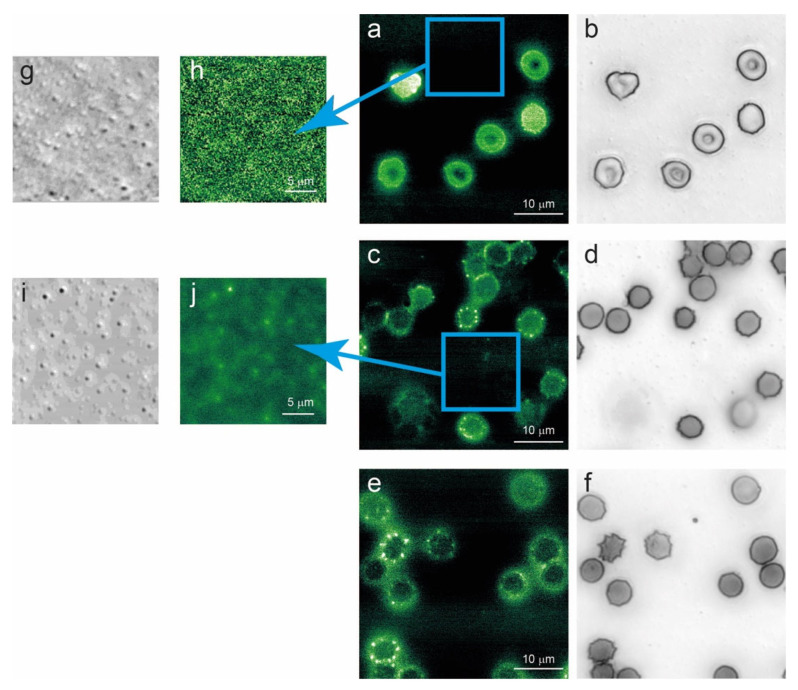
Optical (grey scale) and fluorescence (green false colors) images of G6PD-defective erythrocytes after 1 day (**a**,**b**), 7 days (**c**,**d**), and 12 days (**e**,**f**) of aging in vitro. The presence and the fluorescence of cell-released vesicles are reported at short (**g**,**h**) and intermediate (**i**,**j**) aging times. Along the aging process, a clear alteration in cellular proteins occurs, as a superfluorescence signal localized in spicules and vesicles can be identified in cells with abnormal morphology.

In Figure 5, we show images collected on favism RBCs after 1, 7, and 12 days of aging. Interestingly, the images showed, at intermediate and long aging times, evidence of an accumulation of fluorescent proteins in specific spots of the cell membrane. Namely, the spicules are clearly super-fluorescent, and, in several cases, they are associated with relatively large vesicles released by the cells, but that still adhere to the cell edge.

Interestingly, this fluorescence-related phenomenon occurs as a consequence of alterations associated with aging. Indeed, while the presence of cells with spicules or vesicles can be detected, even at short aging times (especially in favism RBCs) [41], they do not show fluorescence contrasts. Moreover, the micro-vesicles released by the cells on the substrate, as investigated by optical and fluorescence microscopy (Figure 5g,h), show no significant signal at T1, but they are clearly fluorescent at longer aging times.

**Figure 6:**
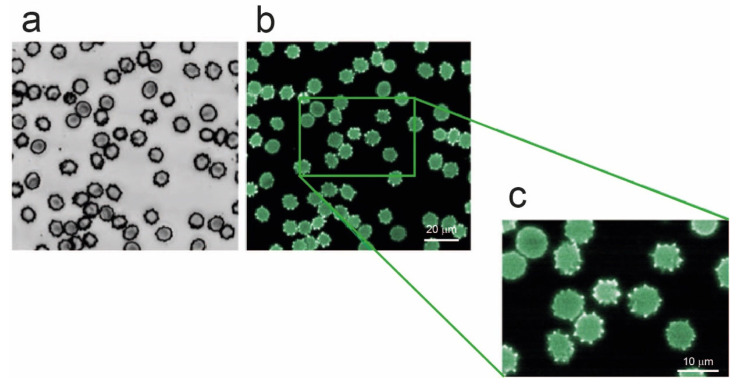
Optical (**a**, grey scale) and fluorescence (**b**,**c**, green false colors) images of erythrocytes depleted of ATP by 24 h incubation at 37 °C. As shown in the magnified panel (**c**), larger fluorescence signal arises from the spikes which appear clearly more brilliant than the rest of the cell.

This interesting phenomenon can be enhanced and associated with aging-related effect by considering the result reported in Figure 6, where RBCs that have been artificially depleted of ATP by 24 h incubation at 37 °C, are shown. This simple treatment is known to accelerate cell metabolism, inducing the consumption of the vast majority of the intracellular ATP [18]. According to the data of Figure 6, energy depletion, which is a typical aging-related phenomenon, is clearly associated with an extensive production of hyperfluorescent spicules.

This result clearly indicates that a pattern of cell alteration, influenced by morphology and strongly involving the Hb, takes place during aging.

## 3. Discussion

The clinical spectrum of favism, although different according to the genetic defect of G6PD, is characterized by severe hemolytic anemias following the contact or ingestion of selected exogen molecules, such as the metabolite of fava beans. At the cellular level, favism erythrocytes have always been considered more fragile than normal cells (namely, with lower mechanical resistance to osmotic stress) and more prone to lysis under various environmental stimuli.

Recently, however, in-depth studies based on the use of the presently employed biological model and focused on the investigation of alteration in the cellular properties during in vitro aging have been carried out. The experimental setup uses RBCs and accelerates their aging rate by incubation in starvation conditions, specifically in the absence of glucose and precursors that allow the cells to replenish ATP using glycolysis. With these premises, the study places both normal and G6PD-defective cells in the same metabolic condition: neither can replenish ATP (or reducing power), and both are forced to use only their initial cellular content. This approach highlights, as the aging increases, intrinsic metabolic differences, including phenomena related to environmental adaptation.

Previous works employing this aging-based approach showed that, in favism RBCs, the main biophysical properties of cells analyzed at T0 (i.e., erythrocytes from fresh blood) are worse than those of the controls [14]. This includes differences at the morphological level (e.g., in the percentage of non-discocytic cells, which is similar to those reported here) and at the structural level (e.g., in the osmotic stress resistance). Surprisingly, however, the defective characteristics in favism RBCs are reduced along their aging, to the point that, in senescent samples, no differences can be observed between healthy and favism erythrocytes, and, in some cases, the healthy RBCs have worse cellular properties. This behavior was explained by considering the cellular intrinsic energy metabolism and, mostly, the ATP consumption over time, which is markedly lower in the favism samples [42]. This causes a modulation of all the ATP-dependent cellular properties and, at medium and long aging times, makes the favism cells even more resilient than the controls.

A deeper investigation focused on the characteristics of ATP production and consumption through a study that linked cellular activity and ATP consumption [43]. In that case, a nanoscale motion sensor was used to measure the cell vibration associated with the metabolic activity of RBCs at different aging times of cells. This experiment was performed on both healthy and favism cells during an ATP reloading process and confirmed an increased resilience of the favism cells as the aging times increased.

The morphological data collected by AFM and reported in this work confirmed that, under the chosen experimental conditions, the aging path of favism and normal RBCs are different. In particular, a larger number of non-discocytic morphologies were observed even in young favism samples and, remarkably, significant production and release of cellular vesicles occur in this sample, especially at intermediate and long aging times.

Besides this pure morphological characterization, in the present work, we investigated the behavior of cellular properties that are certainly connected to the ATP but that are also influenced by the nature of the cells under examination. To this end, we focused on the evolution along the aging of morphometric characteristics and nanomechanical properties of the cells, quantified, respectively, through the membrane roughness and through the stiffness and Young’s modulus. This methodology compares distinct and uncorrelated parameters, collected and mapped onto the same areas of the same cells, to probe with different techniques, the structure and morphological arrangement of the membrane and skeleton.

In a previous study, the behavior of roughness and nanomechanical properties was investigated in erythrocytes from healthy subjects under different environmental conditions [38]. The study demonstrated that, although these parameters exhibit a certain degree of correlation, they do not coincide and do not describe the same biophysical condition of the cells. Here, the investigation is performed on cells from a favism patient, and the data referring to the average values in the samples confirm a certain anticorrelation between Young’s modulus and membrane roughness, where an increase in Y (stiffening) occurring during aging corresponds to a decrease in roughness (reduction in cytoskeletal integrity). However, at intermediate aging times, the population—when analyzed in terms of mean values—was rather heterogeneous with respect to the mean elasticity, but not to the same extent in terms of roughness (Figure 3).

The subsequent analysis was conducted at the single-cell level and highlighted further differences. Indeed, the nanomechanical data showed that, at intermediate aging times, the population contains both “still young” and “already old” cells (producing the large error bar at T7 in Figure 3 right panel) and that the inhomogeneity of these properties can be intrinsic, involving a spatial dispersion of nanomechanical properties on the single-cell surface (see Figure 4j). As the nanomechanical properties are strictly related to the skeletal integrity, this inhomogeneity reflects the instability in the cells’ structure that occurs while erythrocytes are growing senescent. Interestingly, in the specific case, this behavior is not reflected in the roughness, although this parameter is partially sensitive to the structural integrity of the cells as well. Indeed, the membrane arrangement measured by the surface roughness, despite a reduction in its value associated with increased instability of the skeleton network, shows no evidence of locally confined morphometric alteration of the cell surface. A deeper discussion on the physical origin of the differences in the biophysical sensitivity between roughness and Young’s modulus is reported in reference [38].

However, one point remains unclear and concerns the possible source for the transient structural instability that was observed in the cells at the intermediate aging times. To this end, we focused our attention on the possible effects involving Hb and its degradation patterns. Indeed, among the various factors contributing to RBCs’ aging, a predominant role in the case of favism is probably played by the Hb. Due to the genetic characteristics of this pathology, erythrocytes are exposed to greater oxidative stress which, among its various effects, produces a marked increase in Hb oxidation, thereby promoting the onset of many degradative effects. These include the formation of dysfunctional ferric forms, reversible and irreversible hemichromes, protein denaturation, precipitation, and possible binding to band 3 proteins. Overall, these mechanisms can induce morphological alterations and are involved in the vesicle formation, which, in fact, is amplified by the lack of ATP and reducing power that is more pronounced in favism cells than in normal samples [44,45,46].

An indication of the key role of Hb comes from the analysis of the fluorescence microscopy experiments, where the analysis of the cells’ autofluorescence indicates a clear accumulation of self-fluorescent areas at the level of the spicules and into cellular proto-vesicles. Indeed, although the spectroscopic data measured in the regions of increased fluorescence lack enough spectral resolution to directly identify the presence of altered protein fractions, undoubtedly our data shows a clear pattern in which the hyperfluorescent material is localized within proto-vesicles and subsequently encapsulated and expelled from the cells. This phenomenon certainly occurs as a consequence of aging, as released vesicles are not fluorescent when they arise from young cells, but, on the contrary, are clearly fluorescent when investigated at intermediate and long aging times. In this phenomenon the role of Hb is clearly decisive, as the association of the fluorescence detection with the Hb in the vesicle and of the hyperfluorescence with the Hb in the spicules or in proto-vesicles still attached to the mother cell is beyond any doubt. Moreover, hyperfluorescence is clearly an energy-dependent phenomenon. Indeed, an increase in autofluorescence is observed both at advanced stages of aging and in cells that have been artificially depleted from their energetic content, both conditions being characterized by low ATP availability.

In this framework, thus, it seems clear that the collapse of oxidized Hb, by interacting with specific exposed sites (for example on band 3 proteins, which undergo severe rearrangements in the absence of ATP), renders the cytoskeletal structure particularly unstable, thereby contributing to the amplification of the conformational transition intrinsically observed during aging.

This structural instability observed at the intermediate aging times could regard also the formation of vesicles. The production and release in the environment of micro- or even large vesicles are important phenomena that are inherently part of the aging pathway of erythrocytes. Over the years, it has been interpreted in different ways, including as part of an erythrocytes-adapted apoptosis [47,48], but it certainly contributes to the reduction in surface and Hb content that takes place during RBC aging; and the production of vesicles necessarily involves intracellular interaction and a significant rearrangement of the internal skeletal structure [49,50].

In this context, it is worth recalling, that our ultra-morphological AFM data showed that vesiculation is an important phenomenon in the aging of favism samples, as it is much more pronounced and occurs earlier than what is normally observed in healthy individuals. Moreover, our nanomechanical data evidenced that a structural instability occurs during the intermediate and late steps of the aging, when reference data in our experimental conditions indicates that ATP is more massively consumed and that Hb, especially in favism cells, is already significantly oxidized and unstable [19]. As “aged” Hb is poorly functional and its degradation product can be of little use for the cells, they can become potential candidates for release. However, this protein has many additional functions in the cell, and its interaction mechanisms are deeply carved in cell physiology, for instance, in terms of affinity with specific proteins of the skeletal network both in physiological condition and during the formation of Heinz bodies [51,52,53]. In this sense, degraded Hb or even hemichromes can still have preferential interaction and can be precious in modulating certain biological pathways, especially under oxidative conditions [5,54,55].

In this framework, our data suggests that at T7, progressively unstable Hb precipitates in various degradative forms and destabilizes the cell skeleton, contributing to enhancing the formation of spicules and triggering the production of Hb/hemichromes-containing vesicles [56]. This process determines an increased variation in the cell elasticity and, in particular, of its spatial distribution above the cell surface, since the vesicles formation, at this stage, directly (and massively) involves a rearrangement of the membrane skeleton through the junctional complexes and the band 3 proteins. This pattern can be seen clearly in the measurements of elasticity since force curves probe the cell in an extremely local fashion, while the measurement of roughness, arising from a spatial average on 500 nm^2^, simply registers the early stage of this phenomenon cytoskeletal instability as a reduction in skeletal integrity (corresponding to reduction in roughness value).

## 4. Materials and Methods

### 4.1. Sample Preparation

Sample preparation was performed according to a previously employed protocol [14]. In detail, blood samples were obtained from one healthy subject and one favism patient suffering from the Mediterranean variant of the pathology. Blood was collected through venipuncture into Vacutainers (Becton-Dickinson, Franklin Lakes, NJ, USA) and immediately diluted 2-fold in an isotonic, calcium- and glucose-free buffer solution, containing EDTA as anticoagulant (namely, 10 mM sodium phosphate, 140 mM NaCl, EDTA 1 mM) and adjusted to pH 7.4 using NaOH. Then, the samples were immediately centrifuged for 10 min at 3000 rpm at 4 °C. The platelet-rich supernatant and the leukocytes layer deposited on top of the red pellet were removed, while an aliquot of the supernatant plasma was stored at 4 °C and used for the AFM smears. After 4 cycles of re-suspension and washing in the same phosphate buffer, the isolated RBCs were stored under sterile conditions at room temperature (20 ± 1 °C) at 20% hematocrit. A protease inhibitor (phenyl-methyl-sulfonyl-fluoride, 1 mM) was added to the RBC’s solutions to avoid proteolytic degradation during the aging. The entire samples’ incubation (aging) was carried out in the absence of glucose and extracellular calcium, but at the beginning of the experiment, the samples were subjected to an energetic synchronization consisting of an energy depletion followed by immediate reloading. In this way, all the cells start the aging path properly refilled in ATP (and DPG).

The energy reloading procedure was performed, according to a slightly modified De Venuto protocol [57], using a rejuvenation solution, called IPP. This is an isotonic solution of 10 mM inosine, 10 mM pyruvate, 75 mM sodium phosphate, and 23 mM NaCl, with the pH adjusted to 7.4 using NaOH.

Operatively, we depleted the energy of the freshly prepared samples through 24 h incubation at 37 °C in a glucose-free buffer solution. This simple method accelerates cellular metabolism and consumes the most part of their residual ATP (typically 80–90%). After this treatment, the sample was centrifuged for 10 min at 3000 rpm, and the supernatant was substituted by IPP solution in a 1:5 ratio (*v*/*v*) and then incubated again for 3 h at 37 °C. Next, the reloaded RBCs were washed twice and suspended at a final 20% hematocrit in the standard aging buffer. An aliquot of each sample (typically 200 μL) was used immediately before and after the rejuvenation to measure the ATP content and verify the efficiency of the procedure. Typical ATP trends were detailed in previous works [14,18,32] and are not discussed here.

The reloaded samples were maintained in the incubation solution at 20 °C for the entire aging time. After 1, 7, and 12 days of aging, sample smears were prepared in duplicate for AFM analysis. They were prepared by adding a 15 μL aliquot of plasma (stabilized at RT) to the same amount of the sample. The use of plasma warrants the best conservation of cell morphology and a homogeneous dispersion on the glass slide during the smear. After gentle mixing, 6 μL of this plasma and sample solution were manually smeared onto a standard poly-L-lysine-coated glass slide (Thermo Scientific, Menzel-Glaser, Waltham, MA, USA) and finally air-dried under laminar air flow.

In previous works, we verified that properly performed and aseptically stored smears are very stable and remain unmodified over long times.

All experiments and sample storage were carried out at room temperature and 20% relative humidity.

### 4.2. AFM Setup and Data Collection

For all our experiments, we used a Park NX-12 (Park Systems Inc., Suwon, Republic of Korea) AFM. This microscope was mounted on an Olympus IX inverted optical microscope equipped with a high-resolution optical camera. This setup allows correlative analysis by combining optical and AFM imaging of a given area.

We used Bruker DNP-10 AFM cantilevers (Bruker Co., Billerica, MA, USA), with nominal tip size of 10 nm, and employed the sensor with 0.12 N/m nominal elastic constant. Before each experiment, the sensors were calibrated using the thermal-noise routines embedded in the NX-12 software (XEI Data Processing Analysis, v. 5.2) to determine the resonant frequency and the corresponding precise elastic constant of the employed sensor [58].

All the AFM morphological images were collected in air, in soft contact, with a tip-sample interaction below 2 nN and a typical scan rate of 2 rows/s.

**Figure 7:**
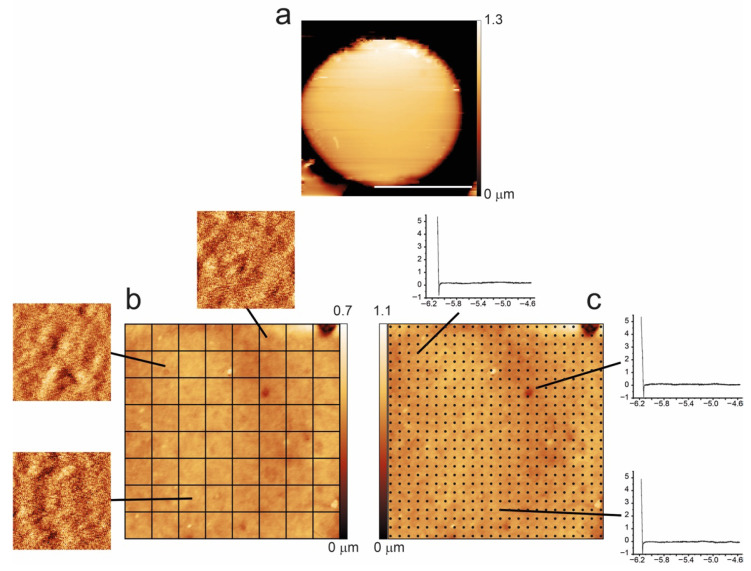
Methodology employed for the data collection of morphometric (roughness) and nanomechanical (Young’s modulus) data. On all selected cells, we collected a high-resolution image (**a**) used for the roughness calculation (performed on the 500 nm sized squared sub-images, **b**), and immediately after, we collected a force volume map (i.e., a matrix of force vs. distance curves) on the very same area (**c**). The scale bar in (**a**) corresponds to 5 μm, while images in (**b**,**c**) are 4 × 4 μm.

In this work, significant effort was dedicated to compare, at the single-cell level, the nanomechanical (Young’s modulus) and the morphometric properties (membrane roughness) of the cells along the aging process. The methodology employed in this analysis is summarized in Figure 7. For every cell, we collected high-resolution images to be used for the measurement of the membrane roughness, possibly on the entire cell surface. To this end, the cell surface was divided into squares of 500 × 500 nm^2^, and every single sub-image was used to measure the roughness. On the very same areas of the very same cells, we also collected maps of force curves, which were used to calculate the nanomechanical properties (i.e., Young’s modulus).

The force curves were collected using the same tip already employed for the morphological measurements, with a tip speed of 10 mm/s and maximum applied force of 4 nN. The typical force curve grid was composed of 40 × 40 or 32 × 32 curves on a 4 × 4 μm^2^ area, ensuring a distance between curves of 100–125 nm.

This methodology for the data collection was repeated on no less than 10 cells per sample to ensure the statistical significance of the data and repeatability of the measurements.

### 4.3. Image Analysis and Roughness Calculation

All AFM images were analyzed using the free software Gwyddion (version 2.59) [59].

In this study, a major effort was dedicated to the measurement of the membrane roughness. The calculation of this parameter has been described in previous works [31,38] and, as shown in Figure 7, requires, for each cell, the division of the high-resolution image (typically 4 × 4 μm^2^ with 1000 or 1200 points) in several sampling areas of square shape and fixed 500 nm sizes. Indeed, roughness has a strong dependence on the area (as well as on the density of points and on the image resolution), and the collection setup must be standardized for comparison. The 4 × 4 μm^2^ images were treated by simple background subtraction followed by plane alignment and were used for the selection of the 500 nm sized sub-images. The processing of each sub-image involved background subtraction, plane alignment, and X- and Y-axis linearization, while all the residual morphological components were removed using a 7th-grade polynomial fit. The choice of the best polynomial fit was assessed in previous works [32], and the use of the 7th-grade polynomial fit was found to provide the best compromise between proper subtraction of disturbing low-frequency (waviness) and preservation of even the tiniest features on the cell membrane.

After this fitting, the surface roughness (*R_rms_*) was measured using the formula:

(1) $$R_{rms}=\;\frac{1}{\left(N-1\right)}\sqrt{\sum_{1}^{N}{\left(X_{i}-X_{m}\right)}^{2}}$$

where *X_i_* is the height of the *i*-th point, *X_m_* is the average height, and *N* is the total number of points.

The many samplings obtained in different areas of a given cell were plotted as single-cell histograms or, alternatively, summarized as a mean cell value. Subsequently, a representative sample roughness value was obtained by averaging the mean values of the various analyzed cells, and this average value was used to compare the behavior of different specimens’ *R_rms_*.

### 4.4. Nanomechanical Properties

The force volume maps collected according to the methodology described in Figure 7 were extracted using Park Systems XEI software (Version 5.2.4 Build 1, Park Systems, Suwon-si, Republic of Korea) to obtain a set of single “.txt” files, which were subsequently analyzed with the freeware software FC_analysis (Version 1.3) [60]. This software allows processing dataset of force curves in a semi-automated way to obtain, from each force curve, the corresponding values of stiffness and elasticity (Young’s modulus). In our experiment, the specific tip–sample interaction required the use of a Bilodeau-modified Sneddon model, which, in our experience, is the best model to study the mechanical properties of RBCs using pyramidal-shaped tips [61,62]. This model, modified using the Bilodeau correction, accounts for the use of a pyramidal indenter. Thus, the indentation profile and the related Young’s modulus can be obtained by modeling the force curves through the following formula:

(2) $$F\;=\frac{2E\delta^{2}}{\pi \left(1-\nu^{2}\right)\tan\alpha }$$

where *F* is the force measured by the AFM, *E* is the Young’s modulus, *δ* is the indentation depth, *ν* is the Poisson’s ratio set as 0.5, and *α* is the tip’s opening half angle (23.5° for the tips we used).

During the collection of each force curve, we ensured that the indentation depth was smaller than 15% of the total height of the cells (i.e., less than 30–40 nm). This ensures that the force curves were not influenced by the substrate properties. The absence of any bottom-effect component is confirmed by the space distribution of the measured values, which is not affected by the substrate in any part of the cell, even in the central biconcavity where the tip–substrate distance is smaller.

### 4.5. Statistical Analysis

All the analyses reported in the present work have been performed by multiple researchers to ensure unbiased interpretation. The data presented has been collected in two independent sample preparations. The statistical analysis was performed using the software Origin Pro (2018, Origin Labs, Northampton, MA, USA). The statistical significance for the average Young’s modulus and the mean roughness were calculated using an ANOVA 1-way test, considering statistical difference significative for *p* < 0.001.

Concerning the statistical size of the dataset (i.e., number of the cells analyzed per sample), the methodology we employed was parameter-based. Namely, for any given parameter (e.g., roughness or Young’s modulus), we collected and analyzed cells up to the point that the addition of new cells in the dataset produces minimal changes in the mean values (assumed as convergence condition). Clearly, this can require larger or smaller number of cells, depending on the samples’ intrinsic characteristics, but, in any case, no less than 10 cells per sample have always been considered.

## 5. Conclusions

The study of the structural alterations occurring during the aging of erythrocytes from favism subjects was carried out using an innovative approach that combines, on the one hand, correlative morphometric and nanomechanical analyses and, on the other, the monitoring of cellular autofluorescence, which is associated with the presence and characteristics of hemoglobin (Hb). From an ultra-morphological perspective, RBC aging in a favism subject differs from that of a healthy individual in terms of a higher occurrence of morphological alterations at the macroscale and a more pronounced development of vesicles at the nanoscale. Moreover, fluorescence analysis revealed the presence of a hyperfluorescence phenomenon with a well-defined spatial and temporal localization: it appears exclusively on cellular spicules and vesicles and only at intermediate–long aging times. Remarkably, vesicles produced and released into the environment by young cells do not exhibit fluorescent properties, whereas those produced at intermediate–long aging times clearly do. This phenomenon is related to time-dependent alterations of Hb and is energy-dependent, since it can be artificially induced, even in fresh cells, by energy depletion. Therefore, in our experimental conditions, it is unequivocally an aging-dependent phenomenon, as ATP availability is known to decrease over time. In parallel, the analysis of membrane roughness and nanomechanical properties highlighted, through different approaches, the existence of a transition at intermediate–long aging times, when the support provided by the cellular skeleton decreases and becomes spatially inhomogeneous.

The overall observed pattern allows the reconstruction of several particularly significant aspects of aging in favism. In this pathology, RBCs are known to have a reduced availability of reducing power and ATP, and maintenance under oxidative stress conditions induces hyper-oxidation and increased structural instability of hemoglobin. Unstable variants of this protein (e.g., hemichromes), upon precipitation, can associate with cytoskeletal proteins and induce nanomechanical and structural instability which, particularly under conditions of ATP depletion, results in the overproduction of abnormal forms and in the incorporation of degraded Hb into macrovesicles (hyperfluorescent) released into the environment.

This mechanism, in addition to providing a comprehensive description of the observed phenomena, prompts reflection on the role of Hb. Indeed, this protein, even in its less functionally active states, seems to play important regulatory role for the cell, and this may also occur while supporting an overall strategy in which an oxidized, poorly functioning, cellular product is eliminated.

Some points remain to be clarified in future experimental approaches concerning the occurrence of altered forms of Hb and their role in accelerating the degradative pathways in favism cells. To this end, high-resolution spectro-microscopy analysis (in different spectral ranges) would be very useful to identify the specific type of protein alterations observed and could be used to fit the molecular mechanism underlying the presently reported observation into the context of the alterations occurring along the aging.

A last remark concerns the methodological approach we employed in this study. Indeed, a direct, point-by-point coupling of morphometric and nanomechanical measurements can provide a deeper understanding of the physical base of complex biological patterns and, more generally, help close the gap between two traditionally distinct methodologies to investigate biosystems. In particular, the present methodological approach is particularly suited to match the current trend in the developments of the AFM technique, which promise to deliver faster and simpler data collection [63]. The implementation of automated morphometric and nanomechanical analysis would transform the present approach from the proof-of-concept shown in the present work to a systematic methodology of data interpretation.

## Data Availability

All data presented in these studies are archived in CNR-ISM repository and will be freely available upon reasonable requests.
